# Acute Coronary Syndrome and Recreational Drug Use: A Comprehensive Review

**DOI:** 10.3390/medicina62081477

**Published:** 2026-07-30

**Authors:** Panagiotis Iliakis, Konstantina Ntalekou, Eleftheria Stamou, Aikaterini-Eleftheria Karanikola, Andreas Mavroudis, Nikolaos Ktenopoulos, Paschalis Karakasis, Panagiotis Theofilis, Obayda Azizy, Anna Pitsillidi, Aikaterini Damianaki, Eirini Beneki, Alexandros Kasiakogias, Christina Chrysohoou, Polykarpos Christos Patsalis, Kyriakos Dimitriadis, Konstantinos Tsioufis

**Affiliations:** 1 First Department of Cardiology, School of Medicine, National and Kapodistrian University of Athens, Hippokration General Hospital, 11527 Athens, Greece; ntalekou95@gmail.com (K.N.); eleftheriastamou1@gmail.com (E.S.); elinakaranikola@gmail.com (A.-E.K.); andreasmavroudis89@gmail.com (A.M.); nikosktenop@gmail.com (N.K.); panos.theofilis@hotmail.com (P.T.); e.beneki@hotmail.com (E.B.); akasiakogias@gmail.com (A.K.); chrysohoou@usa.net (C.C.); dimitriadiskyr@yahoo.gr (K.D.); ktsioufis@gmail.com (K.T.); 2 Division of Cardiology, Angiology and Internal Emergency Medicine, Department of Medicine, Knappschaft Kliniken University Hospital Bochum, Ruhr University Bochum, 44892 Bochum, Germany; dr.azizy@outlook.com (O.A.); polykarpos.patsalis@ruhr-uni-bochum.de (P.C.P.); 3 2nd Department of Cardiology, Ippokrateio General Hospital of Thessaloniki, 54642 Thessaloniki, Greece; pakar15@hotmail.com; 4 Department of Obstetrics and Gynaecology, Rheinland Klinikum Neuss, Preußenstrasse 84, 41464 Neuss, Germany; anna.pitsillidi@gmail.com; 5 Nephrology Department, Hippokration Hospital, 11527 Athens, Greece; aikaterini.damianaki@outlook.com

**Keywords:** acute coronary syndrome, recreational drugs, cocaine, cannabis, amphetamines, myocardial infarction, substance use

## Abstract

Acute coronary syndrome (ACS) remains a leading cause of cardiovascular morbidity and mortality worldwide. Although traditional cardiovascular risk factors remain central to ACS development, recreational drug use is increasingly recognized as a clinically relevant trigger, particularly in younger patients with fewer conventional risk factors. This narrative review synthesized evidence identified through searches of PubMed/MEDLINE and Scopus up to June 2026, including clinical guidelines, systematic reviews, observational studies, mechanistic investigations, and clinically informative case-based evidence. Cannabis, cocaine, amphetamines, methamphetamine, 3,4-methylenedioxymethamphetamine (MDMA), opioids, lysergic acid diethylamide (LSD), synthetic cannabinoids, and polysubstance use may promote myocardial ischemia and infarction through overlapping mechanisms, including sympathetic activation, coronary vasospasm, endothelial dysfunction, oxidative stress, inflammation, platelet activation, thrombosis, arrhythmogenesis, and myocardial oxygen supply–demand mismatch. Clinical presentation may be typical or atypical and may overlap with intoxication, withdrawal, anxiety, neurological symptoms, or non-cardiac chest pain, making diagnosis challenging. Underreporting of recreational drug use is common, and targeted toxicology screening may improve diagnostic accuracy and risk stratification, particularly in patients younger than 50 years, those with few traditional cardiovascular risk factors, or those presenting with otherwise unexplained ST-segment elevation myocardial infarction (STEMI), non-ST-segment elevation myocardial infarction (NSTEMI), coronary vasospasm, arrhythmias, or cardiac arrest. Acute management should generally follow standard ACS guidelines, while considering drug-specific issues such as stimulant-induced vasospasm, sympathetic excess, cautious use of beta-blockers during acute intoxication, and preference for primary percutaneous coronary intervention when fibrinolysis carries increased risk. Long-term care should combine evidence-based secondary prevention with substance-use counseling, addiction medicine referral, cardiac rehabilitation, and behavioural interventions. This review summarizes the pathophysiology, clinical manifestations, epidemiology, treatment considerations, preventive strategies, and knowledge gaps related to recreational drug-associated ACS.

## 1. Introduction

Acute coronary syndrome (ACS) remains a leading cause of cardiovascular morbidity and mortality worldwide and represents a time-sensitive clinical emergency encompassing ST-segment elevation myocardial infarction (STEMI), non-ST-segment elevation myocardial infarction (NSTEMI), and unstable angina [1,2]. Contemporary ACS care relies on early diagnosis, electrocardiographic and biomarker assessment, risk stratification, timely reperfusion or invasive evaluation when indicated, antithrombotic therapy, and intensive secondary prevention [1,2]. Although traditional cardiovascular risk factors such as age, smoking, hypertension, dyslipidemia, diabetes mellitus, chronic kidney disease, and established atherosclerosis remain central to ACS development, non-traditional and behaviour-related triggers are increasingly recognized as clinically relevant contributors to myocardial ischemia and infarction [3,4]. Among these, substance-related exposures are particularly important because they may precipitate ACS through acute hemodynamic, vascular, inflammatory, thrombotic, and arrhythmic mechanisms, even in individuals without advanced pre-existing coronary artery disease [4,5,6,7]. Therefore, recreational drug use should not be regarded as an incidental social history detail, but as a potential ACS trigger, risk modifier, and therapeutic challenge.

Importantly, recreational drug-associated cardiovascular presentations should not automatically be classified as ACS solely on the basis of chest pain, electrocardiographic abnormalities, or elevated cardiac troponin concentrations. According to the Fourth Universal Definition of Myocardial Infarction, acute myocardial injury is defined by a rise and/or fall in cardiac troponin with at least one value above the 99th percentile upper reference limit, whereas myocardial infarction additionally requires evidence of acute myocardial ischemia [8]. Recreational drugs may precipitate type 1 MI through plaque disruption or coronary thrombosis, type 2 MI through coronary vasospasm, severe hypertension, tachycardia, hypoxemia, hypotension, or other supply–demand imbalances, as well as non-ischemic myocardial injury caused by myocarditis, Takotsubo syndrome, direct cardiotoxicity, or associated systemic complications. Spontaneous coronary artery dissection and other non-atherosclerotic coronary disorders should also be considered, particularly in younger patients. Accordingly, the term “drug-associated ACS” should be reserved for presentations in which acute myocardial ischemia has been established, while broader terms such as “drug-associated myocardial injury” should be used when the ischemic mechanism remains unconfirmed.

Recreational drug use is increasingly relevant to cardiology because of its high global prevalence and its disproportionate impact on younger patients. The global burden of drug use disorders has increased substantially over recent decades, with recent estimates suggesting more than 53 million affected individuals worldwide in 2021 and a continuing projected rise over the next 25 years [9]. In the cardiovascular setting, recreational drug use includes a heterogeneous group of agents, such as cannabis and synthetic cannabinoids, cocaine, amphetamines and methamphetamine, 3,4-methylenedioxymethamphetamine (MDMA), opioids, lysergic acid diethylamide (LSD), other psychedelics, and frequent polysubstance use. Although these substances differ substantially in pharmacology, route of administration, duration of effect, and user profile, they may converge on common ACS-relevant pathways, including sympathetic activation, coronary vasospasm, endothelial dysfunction, oxidative stress, inflammation, platelet activation, thrombosis, arrhythmogenesis, and myocardial oxygen supply–demand mismatch [4,5,6,7,10,11]. These exposures are especially important in young patients with ACS, in whom the burden of conventional cardiovascular risk factors may be lower and the contribution of acute triggers may therefore be underestimated. Nevertheless, recreational drug exposure should not automatically be considered the sole cause of ACS in this population, because genetic predisposition, familial hypercholesterolemia, thrombophilic disorders, spontaneous coronary artery dissection, inflammatory conditions, and other non-traditional cardiovascular risk factors may coexist and confound causal attribution [12,13,14].

Drug-associated ACS remains diagnostically and therapeutically challenging because symptoms may overlap with intoxication, anxiety, withdrawal, neurological symptoms, dyspnea, palpitations, altered mental status, or non-cardiac chest pain. In addition, self-reported drug use is often unreliable because of stigma, fear of legal consequences, impaired consciousness, or incomplete history-taking. In the Addiction in Intensive Cardiac Care Units (ADDICT-ICCU) cohort, only 57% of patients with positive toxicology screening disclosed recreational drug use. Although patients with positive tests were younger overall, the published study did not report the specific proportion aged younger than 50 years. These findings nevertheless support targeted toxicology screening in selected patients with ACS, particularly younger individuals, those with few traditional cardiovascular risk factors, or those presenting with otherwise unexplained STEMI or NSTEMI, coronary vasospasm, arrhythmias, or cardiac arrest [15]. Recent multicentre evidence has also reinforced the prognostic relevance of recreational drug use after ACS, showing higher 1-year major adverse cardiovascular events among drug-positive patients, especially those presenting with STEMI [16]. Accordingly, this review summarizes the pathophysiological mechanisms, clinical presentation, epidemiology, acute and long-term management, preventive strategies, and remaining evidence gaps related to recreational drug-associated ACS.

## 2. Literature Search Methodology

A narrative literature review was conducted using PubMed/MEDLINE and Scopus to identify studies addressing the association between recreational drug exposure and ACS. The search included combinations of terms related to acute coronary syndrome, myocardial infarction, myocardial ischemia, coronary vasospasm, coronary thrombosis, and cardiovascular outcomes together with substance-specific terms, including cannabis, synthetic cannabinoids, cocaine, amphetamines, methamphetamine, 3,4-methylenedioxymethamphetamine, opioids, lysergic acid diethylamide, anabolic–androgenic steroids, and polysubstance use. Relevant publications available up to June 2026 were considered.

Eligible evidence included clinical guidelines, scientific statements, systematic reviews and meta-analyses, prospective and retrospective observational studies, registries, mechanistic studies, and clinically informative case series or case reports when higher-level evidence was unavailable. Publications were selected according to their relevance to pathophysiological mechanisms, clinical presentation, epidemiology, diagnosis, acute management, secondary prevention, and long-term cardiovascular outcomes. Studies unrelated to cardiovascular manifestations, articles without sufficient clinical or mechanistic relevance, and duplicate publications were excluded. Reference lists of relevant articles were also reviewed to identify additional eligible studies. Because of substantial heterogeneity in substances, exposure definitions, study populations, outcomes, and study designs, the evidence was synthesized narratively rather than quantitatively.

## 3. Pathophysiology

The cardiovascular effects of recreational drug use are heterogeneous, substance-specific, and frequently overlapping. Although cocaine remains the best-studied agent in the context of ACS, increasing evidence suggests that several other recreational and performance-enhancing substances may contribute to myocardial ischemia and infarction. The principal mechanisms include sympathetic nervous system activation, coronary vasospasm, endothelial dysfunction, oxidative stress, inflammation, platelet activation, thrombosis, accelerated atherosclerosis, arrhythmogenesis, and myocardial oxygen supply–demand mismatch. These mechanisms may act acutely, as in stimulant-induced vasoconstriction or thrombosis, or chronically, through vascular injury, inflammatory activation, and atherosclerotic progression.

### 3.1. Cannabis

Despite widespread cannabis use, the mechanisms linking cannabis exposure to increased cardiovascular risk remain incompletely defined. Cannabis, derived from *Cannabis sativa*, contains more than 60 bioactive compounds, with Δ-9-tetrahydrocannabinol (THC) being the primary psychoactive constituent [7]. THC exerts its effects through the endocannabinoid system by acting as a mixed agonist at cannabinoid receptor type 1 (CB1) and cannabinoid receptor type 2 (CB2). CB1 receptors are predominantly expressed in the central nervous system but are also present in peripheral tissues, including the heart, adrenal glands, and adipose tissue, whereas CB2 receptors are mainly involved in immune regulation [17]. Emerging in vivo and in vitro evidence suggests that CB2 signaling may influence the initiation and progression of atherosclerotic plaque formation [18].

Sympathetic nervous system activation is a key mediator of cannabis-related cardiovascular effects. THC-induced norepinephrine release may cause tachycardia, increased blood pressure, and reduced left ventricular ejection fraction, thereby increasing myocardial oxygen demand in a dose-dependent manner. At the same time, cannabis smoking may increase carboxyhaemoglobin levels and promote coronary vasospasm, reducing myocardial oxygen supply and predisposing to ischemia [19]. Cannabis may also induce oxidative stress through increased reactive oxygen species generation, thereby contributing to endothelial dysfunction and vascular injury [17,20,21].

Several case reports have linked cannabis use to transient myocardial ischemia due to microvascular or epicardial coronary artery spasm, probably reflecting the complex vasoconstrictive effects of THC [21,22,23,24]. These effects may be more pronounced with synthetic cannabinoids, which have longer half-lives and greater potency and may increase intracellular calcium in cardiac and vascular tissues, promoting vasoconstriction and ischemia [20,25]. Myocardial infarction (MI) may occur when haemodynamic stress triggers disruption of vulnerable atherosclerotic plaques, resulting in thrombus formation [23]. Although platelets synthesize endogenous cannabinoids, evidence regarding the effects of THC on platelet function remains conflicting [7,23,26]. Moreover, findings from in vitro experiments should be interpreted cautiously, because the concentrations used may not reflect clinically achieved circulating levels, while extensive protein binding, rapid tissue distribution, hepatic metabolism, and formation of active or inactive metabolites may substantially modify the in vivo effects of THC on platelet and coagulation pathways [10,27,28]. THC has been shown to enhance procoagulant activity by prolonging lipopolysaccharide-stimulated tissue factor expression in activated monocytes and increasing platelet activation markers, including glycoprotein IIb/IIIa and P-selectin [27,28]. These mechanisms may explain reports of thrombotic coronary occlusion in young individuals without underlying atherosclerosis [23,26,29]. Overall, the acute cardiovascular effects of cannabis are predominantly related to sympathetic activation, tachycardia, increased myocardial oxygen demand, coronary vasospasm, and possible thrombosis, whereas chronic exposure may contribute to persistent endothelial dysfunction, oxidative stress, inflammatory activation, and progression of atherosclerotic vascular disease [10,17,21].

### 3.2. Cocaine and Amphetamines

Cocaine may cause myocardial ischemia and infarction through several complementary mechanisms. By blocking norepinephrine and dopamine reuptake, it increases catecholamine availability and sympathetic nervous system activation, leading to tachycardia, hypertension, and myocardial oxygen supply–demand mismatch. Cocaine also promotes coronary vasoconstriction through α-adrenergic stimulation and increased endothelin-1 levels. Its prothrombotic effects include increased platelet count, activation, and aggregation, as well as elevated levels of plasminogen-activator inhibitor, von Willebrand factor, and fibrinogen. Autopsy studies have also linked cocaine use to early coronary atherosclerosis and thrombosis [5]. In a recent post-mortem study, greater inflammatory cell infiltration was observed in myocardium from cocaine-related deaths compared with controls, implicating inflammation in cocaine-induced myocardial injury [30]. Cocaine may also promote immune activation through reduced anti-inflammatory markers and increased pro-inflammatory mediators, including tumor necrosis factor-α (TNF-α) and interleukin-6 (IL-6), contributing to vascular and myocardial injury [31]. The relative importance of these mechanisms may vary according to the pattern and duration of exposure. Acute use is more strongly associated with catecholamine excess, coronary vasoconstriction, thrombosis, and myocardial oxygen supply–demand mismatch, whereas chronic use may contribute more substantially to endothelial injury, inflammation, accelerated atherosclerosis, and structural myocardial damage. Although cardiovascular events have been reported with intranasal, smoked, intravenous, and oral exposure, comparative evidence is insufficient to determine whether specific routes independently modify the contribution of these mechanisms, particularly because dose, purity, frequency of use, and polysubstance exposure are often incompletely characterized [5,12,30,31].

Amphetamine-type stimulants and cathinones may also be associated with ACS, although the available evidence remains limited and largely observational. Interpretation is further complicated by the high prevalence of polysubstance use among methamphetamine users, including concurrent exposure to cocaine, cannabis, opioids, alcohol, and other stimulants, which may produce overlapping or synergistic cardiovascular effects and limit causal attribution to a single agent [11,15]. These compounds can produce cardiovascular events similar to those observed with cocaine, mainly through sympathoadrenergic stimulation, vasospasm, hypertension, tachycardia, oxidative stress, and direct myocardial toxicity. MDMA, commonly known as ecstasy, has amphetamine-like properties and may cause cardiotoxicity, although its precise cellular effects remain incompletely defined. The main proposed mechanism of MDMA-induced MI is coronary vasospasm caused by sympathoadrenergic stimulation. MDMA increases serotonin, norepinephrine, and dopamine either by blocking their reuptake or by promoting their release. At high concentrations, MDMA metabolites may generate reactive oxygen and nitrogen species, leading to lipid peroxidation, mitochondrial DNA damage, calcium overload, oxidative stress, and ultimately cell injury or death [32,33,34]. Regarding ketamine, reported acute complications are most often related to agitation, aggression, trauma, or risk-taking behaviours driven by dissociative effects. Although interpretation is complicated by frequent polysubstance use, MI is not generally recognized as a direct consequence of acute ketamine toxicity [35].

### 3.3. LSD

LSD interacts with serotonergic, dopaminergic, and adrenergic receptors in both the central nervous system and peripheral tissues. Its cardiovascular effects appear to be mediated mainly by central sympathetic stimulation through 5-HT2A receptor activation, which may lead to tachycardia, increased blood pressure, and hyperthermia. In the periphery, LSD has been reported to antagonize 5-HT2A receptors, which could theoretically influence platelet aggregation and atherosclerosis, although clinical evidence remains limited [36]. Overall, the long-term cardiovascular impact of LSD remains uncertain. Evidence linking LSD specifically to ACS is derived predominantly from isolated case reports rather than case series or dedicated cardiovascular cohorts. These reports suggest that sympathomimetic activation and vasoconstriction may rarely contribute to myocardial ischemia, ACS, or cerebrovascular events [37]. Larger epidemiological studies specifically quantifying the incidence or relative risk of LSD-associated ACS or MI are currently lacking, while controlled clinical studies have mainly demonstrated transient increases in heart rate and blood pressure without establishing a clear association with major cardiovascular events [36,38].

### 3.4. Opioids

Opioids act through G-protein-coupled receptors and activate multiple signaling pathways, including mitogen-activated protein kinases, regulation of adenylyl cyclase, and modulation of ion conductance. The classical opioid receptors—μ (MOR), δ (DOR), and κ (KOR)—are widely distributed in the nervous system [39]. Opioids primarily influence cardiovascular physiology through effects on the vasomotor center, causing hypotension, bradycardia, and, in some settings, QT prolongation. Heroin is a short-acting opioid, and its use is frequently associated with major cardiovascular complications such as embolic stroke and infective endocarditis [39,40].

The relationship between opioids and atherosclerotic cardiovascular disease is complex. Chronic opioid use has been associated with inflammation, reactive oxygen species generation, microvascular dysfunction, metabolic and hormonal dysregulation, and atherosclerosis progression. For example, elevated interleukin-1 levels have been observed in opioid users, supporting a possible inflammatory contribution to vascular injury. In contrast, acute opioid receptor activation may exert cytoprotective effects. Acute KOR activation in human umbilical vein endothelial cells reduces intracellular reactive oxygen species levels, while acute DOR activation induces the PI3K/Akt pathway and promotes cell survival under hypoxic or ischemic conditions. However, these potentially protective effects appear to be attenuated with chronic exposure because of receptor desensitization and reduced downstream signaling [4,40,41].

Opioid withdrawal has distinct cardiovascular implications. In contrast to opioid intoxication, withdrawal is characterized by heightened catecholaminergic activity, resulting in tachycardia, hypertension, and an abrupt increase in myocardial oxygen demand. These physiological changes may destabilize high-risk individuals, particularly those with advanced coronary artery disease, severe valvular heart disease, or significant left ventricular systolic dysfunction. Importantly, withdrawal-associated cardiovascular events may also occur during hospitalization, medically supervised detoxification, or abrupt interruption of chronic opioid therapy, making appropriate recognition, monitoring, and symptom control clinically relevant [39].

### 3.5. Other Performance-Enhancing Substances

Although not always categorized together with classical recreational drugs, anabolic–androgenic steroids represent an important substance-related cardiovascular exposure, particularly among young and physically active individuals. Their inclusion in the present review is clinically relevant because these agents may increase susceptibility to myocardial ischemia and acute coronary events through mechanisms that overlap with those of other recreational substances. Chronic supraphysiological anabolic–androgenic steroid use has been associated with hypertension, adverse lipid remodeling, endothelial dysfunction, oxidative stress, myocardial hypertrophy, fibrosis, arrhythmias, cardiomyopathy, accelerated atherosclerosis, and sudden cardiac death. These effects may promote coronary atherosclerotic progression, increase thrombotic tendency, and worsen myocardial oxygen supply–demand mismatch in the setting of pathological cardiac remodeling. Importantly, anabolic–androgenic steroids are frequently used together with other performance-enhancing substances, including growth hormones, stimulants, thyroid hormones, and diuretics, and such combinations may produce additive or synergistic cardiovascular toxicity [42].

## 4. Clinical Manifestations

Recreational drug use is common worldwide, with an estimated 316 million individuals reporting use within the previous year; this broader population is distinct from the smaller subgroup of more than 53 million individuals estimated to have a drug use disorder. Substances such as cannabis, cocaine, amphetamines, MDMA, and opioids are among the most commonly implicated [15]. In patients with drug-induced ACS, clinical presentation may be either typical or atypical and often varies according to the substance involved. Although many patients present with chest pain, jaw or epigastric discomfort, fatigue, dyspnea, palpitations, diaphoresis, or sudden cardiac arrest, these symptoms are not specific and may overlap with intoxication, withdrawal, psychiatric disorders, or other non-cardiac conditions [8,43].

When acute myocardial injury is identified after recreational drug exposure, the diagnostic evaluation should establish whether the underlying mechanism is ischemic and, if so, distinguish plaque-related type 1 MI from coronary thrombosis without major atherosclerosis, vasospasm, spontaneous coronary artery dissection, or supply–demand mismatch. Coronary angiography remains central in patients with suspected ACS who meet guideline-based criteria for an invasive strategy, as it may identify obstructive coronary disease, acute thrombotic occlusion, dissection, or angiographically normal or near-normal coronary arteries [1]. When the angiographic findings are inconclusive, intracoronary imaging with optical coherence tomography or intravascular ultrasound may be considered selectively to detect plaque rupture or erosion, residual thrombus, intramural hematoma, or other non-atherosclerotic coronary pathology [1]. Transthoracic echocardiography provides rapid assessment of regional wall-motion abnormalities, left ventricular systolic function, and alternative structural diagnoses. Cardiac magnetic resonance is particularly valuable when unobstructed coronary arteries or an atypical clinical course raise suspicion of myocarditis, Takotsubo syndrome, or non-ischemic toxic myocardial injury, because it can characterize myocardial oedema, scar, and the distribution of tissue injury [1,44]. In selected clinically stable patients with persistent or recurrent symptoms and no obstructive coronary disease, coronary functional assessment may help identify epicardial vasospasm or coronary microvascular dysfunction [45,46]. These investigations should be individualized according to clinical stability, angiographic findings, and the suspected underlying mechanism. The principal differential diagnoses, their typical angiographic and imaging features, and their main management implications are summarized in Table 1.

Diagnosing ACS in patients with recent recreational drug exposure remains a significant clinical challenge. Many affected patients are young and lack traditional cardiovascular risk factors, which can lower clinical suspicion for ACS and delay recognition of potentially life-threatening ischemia. Furthermore, individuals may be reluctant to disclose recreational drug use because of stigma or fear of legal consequences, making accurate history-taking more difficult. Symptoms such as anxiety, agitation, dyspnea, nausea, palpitations, or altered mental status are often initially attributed to intoxication or withdrawal rather than myocardial ischemia, resulting in delays in electrocardiography, cardiac biomarker assessment, and definitive treatment. In addition, electrocardiographic abnormalities caused directly by stimulant toxicity, tachyarrhythmias, or electrolyte disturbances may further complicate interpretation and make differentiation from true ischemic changes particularly challenging.

Cocaine use is strongly associated with acute cardiovascular complications and may precipitate ACS through coronary vasospasm, thrombosis, endothelial injury, and increased myocardial oxygen demand. Cocaine produces dose-dependent increases in heart rate and blood pressure, while common accompanying symptoms include dyspnea, anxiety, palpitations, dizziness, nausea, and diaphoresis. Dyspnea and diaphoresis are particularly frequent, occurring in approximately 60% and 40% of patients, respectively. Although many individuals present with classic ischemic chest pain, atypical manifestations such as pleuritic chest pain, syncope, vomiting, nausea, or isolated palpitations are also common and may obscure the diagnosis. Patients with suspected cocaine toxicity may additionally exhibit hypertension, altered mental status, seizures, headaches, paranoia, or focal neurologic deficits, further complicating clinical evaluation and sometimes mimicking neurologic or psychiatric emergencies rather than myocardial ischemia [47]. MI most commonly develops within the first few hours after cocaine use, although delayed presentations have also been reported. A case-crossover study demonstrated that the risk of MI increases approximately 24-fold during the first hour following cocaine consumption, declining sharply thereafter to a fourfold increase during the subsequent two hours. The interval between cocaine use and onset of chest pain may be as short as 60 min in many patients, although symptoms can occasionally appear much later, with reported median delays of up to 18 h [5,48]. Pathological studies have demonstrated significant coronary artery disease even in relatively young cocaine users, with one large autopsy study of cocaine-related sudden deaths reporting significant epicardial coronary artery disease in 28% and small-vessel disease in 42% of cases despite a mean age of only 34 years [49].

Cannabis use appears to represent a significant and independent risk factor for acute cardiovascular events, even in individuals without traditional cardiovascular risk factors. Increasing evidence suggests that cannabis may precipitate ACS and MI, particularly within the first hour after consumption, during which the risk of MI may increase nearly fivefold. This association is especially notable in young and otherwise healthy individuals presenting with chest pain [50]. In addition to typical ischemic symptoms, cannabis use may also cause anxiety, confusion, disorientation, sedation, and tachycardia, which can complicate clinical assessment and delay recognition of underlying myocardial ischemia [10].

Methamphetamine and amphetamine use are associated with an increased risk of coronary artery disease and MI. Although methamphetamine users may demonstrate lower rates of obesity or hypercholesterolemia due to the drug’s appetite-suppressing effects, these substances promote atherosclerosis through endothelial dysfunction, inflammation, and vascular injury [11]. Chronic cardiovascular complications most commonly include coronary artery disease and cardiomyopathy. Acute presentations may be severe, including STEMI, ventricular fibrillation, and cardiac arrest, even in otherwise healthy young patients. Importantly, amphetamine-related MI may also present atypically or silently, particularly in older or diabetic patients, with minimal symptoms and initially normal cardiac biomarkers despite significant coronary occlusion [51,52].

MDMA possesses amphetamine-like properties and commonly produces tachycardia, hyperthermia, and excessive sweating through intense sympathetic nervous system activation. Cardiovascular complications are primarily related to increased catecholamine release and may include arrhythmias, asystole, and cardiovascular collapse. Although MI is less common with MDMA than with other stimulants, cases of ACS and myocardial injury have been reported. Similar to cocaine and amphetamines, MDMA may induce coronary vasospasm, leading to chest pain, ischemic electrocardiographic changes, and, in severe cases, MI [11,53].

Heroin and other opioids may contribute to major adverse cardiovascular events during both overdose and withdrawal through hemodynamic, vascular, and proarrhythmic effects. Acute opioid receptor-mediated cardiovascular manifestations include hypotension, orthostatic symptoms, syncope, and bradycardia, largely resulting from vasodilation mediated by μ-receptor activation. These effects are frequently accompanied by peripheral oedema, flushing, and palpitations. A large nested case–control study involving 1.7 million opioid users reported a 1.28-fold increased risk of MI compared with nonusers. Furthermore, a retrospective claims analysis of chronic opioid users identified markedly increased rates of arrhythmias, acute heart failure, stroke, STEMI, and NSTEMI, with these cardiovascular events associated with substantially higher mortality [39].

## 5. Epidemiology and Risk Factors of Drug-Related ACS

### 5.1. Incidence of ACS Associated with Recreational Drug Use, Age, and Demographic Considerations

Substance use has been increasing globally, making it an important contributor to cardiovascular disease (CVD) risk and outcomes. The profile of drug-related ACS is unique, as affected patients are commonly younger, predominantly male, active smokers, and have fewer traditional cardiovascular risk factors [12,13,54,55]. Recreational use of cannabis, amphetamines, and other drugs was independently linked to premature CVD in the nationwide Veterans Affairs Healthcare database and the Veterans with Premature Atherosclerosis (VITAL) registry [14]. Patients with substance use exhibit higher rates of STEMI and tend to have more severe disease, despite a lower prevalence of diabetes, hypertension, and dyslipidemia [13,54].

According to a recent multicenter observational study, the most frequently detected drugs in Intensive Cardiac Units were cannabis (9.1%), opioids (2.1%), cocaine (1.7%), and amphetamines (0.7%) [15]. Among patients < 50 years old with MI, cocaine and/or marijuana use is present in approximately 10% and is associated with worse prognosis [3,43]. However, among patients with recent cocaine use hospitalized with chest pain, the actual incidence of MI remains approximately 6%, with lower incidence reported in non-urban populations or older age groups [5,56]. Similarly, a retrospective analysis of the Partners Young Myocardial Infarction (YOUNG-MI) registry reported cocaine use, marijuana use, or both in 4.7%, 6.0%, and 1.7% of patients with type 1 MI, respectively [13]. Although evidence on the cardiovascular effects of opioid use remains limited, several studies support an association with increased cardiovascular risk [39,57]. A large nested case–control study by Li et al. highlighted increased MI risk among users of morphine (odds ratio [OR] 1.71), meperidine (OR 2.15), and opioid polytherapy (OR 1.46) for noncancer pain [58]. In contrast, LSD-associated ACS appears extremely rare, with cardiovascular events representing a negligible proportion of LSD-related adverse events and no robust epidemiological data quantifying LSD-associated ACS or MI risk [59,60]. Of note, only 57% of patients with positive drug screens disclosed substance use, supporting the importance of toxicology screening in selected younger patients with ACS and few risk factors [15,61].

### 5.2. Longitudinal Risk

People who use methamphetamine show a 41% increased hazard of subsequent acute MI compared with matched controls, with the highest risk occurring in very young users aged 15–34 years, whereas people who use cocaine have a 25% increased risk according to a large retrospective cohort study [62].

### 5.3. Synergistic Effects

Multiple drug detection occurs in 28% of patients with positive drug screens and is associated with worse outcomes. An OR of 12.7 for in-hospital major adverse events was reported in patients with poly-drug use compared with 8.84 in those with single-drug use [15]. Concurrent use of cocaine with other psychotropic substances, such as alcohol and/or heroin, significantly increases cocaine blood levels by up to 30%, causing increased and prolonged cardiovascular risk [63]. Co-use of cocaine and cannabis also appears to increase CVD risk [30,64]. The combination of intranasal cocaine use and smoking has particularly adverse effects in patients with pre-existing coronary artery disease, resulting in marked supply–demand mismatch and exacerbated coronary vasoconstriction of diseased segments [65,66]. In contrast, the synergistic effect of opioid use and traditional cardiovascular risk factors remains unclear [39].

### 5.4. Patterns of Use

Both acute and chronic stimulant use increase ACS risk, but through different pathways. Acute use triggers events through sympathetic activation, coronary vasospasm, and thrombosis, while chronic use accelerates atherosclerosis and inflammation [12,67]. MI risk appears independent of the route of administration for stimulants [68]. Although cocaine can increase ACS risk acutely, particularly within the first hour, symptoms may occur several hours after cocaine ingestion, when blood concentrations are low or even undetectable [5]. Chronic cocaine use may also induce atherosclerosis [12,56]. Regarding opioid use, the correlation with MI predominantly arises from prolonged cumulative exposure and appears dose-dependent [57].

### 5.5. Outcomes

In hospitalized patients, recreational drug use is associated with worse prognosis. Higher rates of major adverse events, including death, resuscitated cardiac arrest, and cardiogenic shock, have been reported [15]. However, the association between marijuana use and mortality in MI survivors remains controversial. Although an earlier prospective cohort study found no conclusive association between marijuana smoking and mortality, limitations such as residual confounding, self-report bias, secondary prevention bias, loss to follow-up, and low prevalence of marijuana use suggest that increased mortality among marijuana users cannot be excluded [69]. More recent evidence indicates that cocaine and/or marijuana use in MI patients is associated with increased adverse events [3,13,43]. Such patients were slightly less likely to undergo cardiac catheterization and, when needed, coronary artery bypass grafting, although no significant differences were observed in rates of coronary revascularization [13,70]. Patients with substance use also had higher rates of out-of-hospital cardiac arrest, all-cause mortality, and cardiovascular mortality compared with patients without substance use, with associations remaining significant after adjustment for confounders [13]. An unfavourable prognosis has similarly been reported among STEMI patients with opioid use disorder, with higher mortality (7.4% vs. 4.3%), cardiogenic shock (11.7% vs. 7.9%), and in-hospital cardiac arrest compared with non-users [70]. Adverse prognosis in amphetamine-related MI was also highlighted by a large systematic review, with mortality rates of 7% in NSTEMI and 14% in STEMI patients. Interestingly, young age appears to modify risk estimation in methamphetamine users, perhaps because of bias, different use patterns, or cessation of use at older age [32,71].

## 6. Treatment

### 6.1. Acute Management

#### 6.1.1. Standard ACS Protocols and Drug-Specific Considerations

Drug-induced ACS should generally be managed according to standard ACS guidelines, with additional attention to drug-specific mechanisms such as coronary vasospasm, catecholamine excess, hypertension, thrombosis, and myocardial oxygen supply–demand mismatch. In the prospective multicentre ADDICT-ICCU study, recreational drug use was detected in 11% of patients admitted to intensive cardiac care units and was independently associated with a significantly increased risk of in-hospital major adverse cardiovascular events (adjusted OR 8.84, 95% confidence interval (CI) 4.68–16.7; *p* < 0.001) [15].

Standard ACS management remains the foundation of care. In STEMI, primary percutaneous coronary intervention (PCI) is the preferred reperfusion strategy whenever available, while fibrinolysis is reserved for cases in which timely PCI cannot be achieved. In NSTE-ACS, an immediate or early invasive strategy should be guided by clinical risk. Pharmacological treatment includes dual antiplatelet therapy with aspirin and a P2Y12 receptor inhibitor, parenteral anticoagulation during the acute phase, anti-ischaemic therapy, and comprehensive secondary prevention after stabilization. Intravenous opioids may be used for severe persistent chest pain, and intravenous beta-blockers may be considered in haemodynamically stable patients with ongoing ischemia, hypertension, or tachycardia, provided that acute heart failure, cardiogenic shock, atrioventricular conduction disease, or other contraindications are absent. Following PCI, dual antiplatelet therapy is generally continued according to guideline-based ischemic and bleeding risk assessment [1,2]. In patients presenting with myocardial infarction with non-obstructive coronary arteries (MINOCA), antiplatelet treatment should be individualized according to the identified underlying mechanism, because plaque disruption, coronary thrombosis, vasospasm, spontaneous coronary artery dissection, and non-ischemic mimics may have different therapeutic implications [72].

The main guideline-based recommendations relevant to drug-associated ACS and the practical acute management considerations for stimulant-associated presentations are summarized in Table 2 and Table 3. Cocaine- and methamphetamine-associated ACS requires specific attention because vasospasm and sympathetic overstimulation may dominate the clinical presentation. Cocaine may cause ACS through coronary vasospasm, dissection, thrombosis, positive chronotropic and hypertensive effects, and direct myocardial toxicity, while methamphetamines may cause similar cardiovascular complications. Urine toxicology screening should be considered when substance use is suspected as a cause of, or contributor to, ACS, especially in younger patients [5]. Specific recommendations remain limited and are largely based on the 2014 AHA/ACC NSTE-ACS Guidelines. These guidelines support standard ACS treatment but emphasize early use of benzodiazepines, alone or in combination with nitroglycerin, for hypertension, tachycardia, anxiety, and sympathetic excess. Calcium channel blockers may be considered when ischemic symptoms or vasospasm persist despite nitrates and benzodiazepines [73].

Fibrinolytic therapy should be used with caution in cocaine-associated ACS. Cocaine users may present with severe hypertension, aortic dissection, seizures, recent trauma, or other conditions that increase bleeding risk, while cocaine itself may cause vascular injury and sudden increases in blood pressure. Consequently, thrombolytic therapy may carry a higher risk of intracranial hemorrhage, making primary PCI the preferred reperfusion strategy whenever available [74]. Finally, in selected coronary anatomies, particularly small-vessel disease, in-stent restenosis, or situations in which avoiding a permanent metallic scaffold may be desirable, drug-coated balloon angioplasty has emerged as a potential PCI strategy. However, its role in ACS and drug-associated ACS should be individualized according to lesion morphology, thrombotic burden, and guideline-based interventional judgment [75].

#### 6.1.2. β-Blockers in Stimulant-Associated ACS

The use of β-blockers in patients with cocaine- or methamphetamine-associated ACS remains controversial. Traditionally, β-blockers were avoided because blockade of β-adrenergic receptors during persistent catecholamine excess was thought to cause unopposed α-adrenergic stimulation, potentially leading to severe hypertension, coronary vasoconstriction, and worsening myocardial ischemia [76]. This concern formed the basis of recommendations against acute β-blocker administration during active cocaine intoxication [73,76]. During active stimulant intoxication with severe sympathetic excess, hypertension, tachycardia, or suspected coronary vasospasm, benzodiazepines and nitrates should remain the preferred initial treatment, with calcium-channel blockers considered when ischemia or vasospasm persists [73]. Evidence questioning the clinical relevance of unopposed α-adrenergic stimulation does not establish that intravenous β-blockers should be administered routinely during ongoing intoxication [73,76]. However, the clinical evidence supporting this hypothesis is limited, and more recent data have questioned its relevance in routine practice. A meta-analysis of patients with cocaine-associated chest pain found no increase in adverse cardiovascular outcomes among those treated with β-blockers compared with those who did not receive β-blockers [77]. Similarly, observational studies suggest that β-blocker therapy may be safe and potentially beneficial in selected patients. In the RUTI-Cocaine Study, which included 57 patients with cocaine-associated ACS, β-blocker treatment was associated with lower rates of major adverse cardiovascular events, all-cause mortality, and cardiovascular mortality during follow-up [78]. Rangel et al. also reported that β-blocker therapy was not associated with increased in-hospital adverse events, while discharge β-blocker therapy was associated with lower long-term cardiovascular mortality (hazard ratio (HR) 0.29, 95% CI 0.09–0.98; *p* = 0.047) [79].

Given their additional α-blocking properties, labetalol and carvedilol have been proposed as potentially safer alternatives when β-blockade is considered necessary [80]. In patients with persistent hypertension or tachycardia despite initial treatment, a combined α/β-blocking agent, particularly labetalol, may be considered selectively when the patient is haemodynamically stable and ongoing vasospasm or acute heart failure is not present [73,80]. In contrast, evidence regarding β-blocker use in methamphetamine-associated ACS is even more limited and is largely extrapolated from cocaine studies [76]. Therefore, routine β-blocker administration during acute stimulant intoxication remains discouraged, but selected use after stabilization may be reasonable when clinically indicated, particularly with agents that also provide α-blockade. After the acute stimulant effect has resolved and the patient is clinically stabilized, β-blocker therapy may be considered according to conventional indications, including previous myocardial infarction, reduced left ventricular ejection fraction, hypertension, or clinically significant arrhythmia [1,2]. Concerns derived from the acute intoxication setting should not lead to indefinite withholding of evidence-based chronic β-blocker therapy in patients with a clear indication [1,2,77,78,79,80]. Evidence in methamphetamine-associated ACS remains substantially more limited than that for cocaine and is largely extrapolated from cocaine-related studies; therefore, direct equivalence between the two substances should not be assumed [76]. In patients with reduced left ventricular ejection fraction and concern regarding recurrent stimulant exposure, carvedilol may be a reasonable option because of its combined α- and β-blocking properties [80]. Prospective randomised studies are needed to clarify timing, agent selection, and patient subgroups most likely to benefit.

### 6.2. Long-Term Management

#### 6.2.1. Secondary Prevention and Substance-Use Counseling

Long-term management of drug-induced ACS should combine standard secondary prevention with interventions targeting ongoing substance use. Current guidelines emphasize comprehensive risk factor management, including optimization of blood pressure, lipid levels, glycemic control, body weight, physical activity, diet, sleep quality, smoking cessation, and avoidance of harmful substances such as alcohol, cocaine, amphetamines, and other recreational drugs [1,81]. Early and intensive lipid-lowering therapy after ACS remains a cornerstone of secondary prevention, with contemporary evidence supporting rapid LDL-C reduction and escalation beyond statins to ezetimibe, PCSK9 inhibitors, and emerging lipid-targeted therapies when appropriate [82,83].

Referral to specialized addiction services should be considered when appropriate. This is supported by recent data showing that recreational drug use was independently associated with higher 1-year major adverse cardiovascular events (adjusted HR 2.70, 95% CI 1.30–5.57; *p* = 0.013), with an even stronger association among STEMI patients (adjusted HR 4.11, 95% CI 1.60–10.5; *p* = 0.005) [16]. These findings support routine assessment of substance use and multidisciplinary follow-up as integral components of long-term care after drug-induced ACS.

#### 6.2.2. Medication Interactions and Adherence Challenges

Medication adherence and drug interactions represent important challenges during long-term follow-up of patients with drug-induced ACS. Morphine administration has been associated with lower plasma concentrations and delayed antiplatelet effects of oral P2Y12 inhibitors, particularly ticagrelor, in patients with acute MI [84]. In addition, methadone has been associated with QT interval prolongation and may further increase arrhythmic risk when administered concomitantly with other QT-prolonging medications [39]. Beyond pharmacological interactions, maintaining adherence to secondary prevention therapies represents an important challenge in this population. Patients with substance use disorders constitute a particularly high-risk group, given their increased cardiovascular burden and risk of recurrent adverse events [16,85]. These findings support the need for close follow-up, regular reassessment of substance use, and reinforcement of adherence to evidence-based secondary prevention therapies after hospital discharge.

#### 6.2.3. Sudden Cardiac Death Risk and Wearable Cardioverter-Defibrillator

Drug-associated ACS and related myocardial injury may be complicated by ventricular fibrillation, sustained ventricular tachycardia, cardiac arrest, extensive myocardial damage, or substantially reduced left ventricular ejection fraction. In some patients, however, the arrhythmic substrate may be transient or potentially reversible following coronary revascularization, abstinence from the offending substance, correction of electrolyte and metabolic abnormalities, resolution of acute myocardial stunning or inflammation, and optimization of guideline-directed medical therapy. Consequently, immediate implantation of an implantable cardioverter-defibrillator may be inappropriate or premature, particularly during the conventional waiting periods following MI or a new diagnosis of left ventricular systolic dysfunction.

In carefully selected patients with clinically meaningful but potentially transient risk of sudden cardiac death, a wearable cardioverter-defibrillator may provide temporary protection while left ventricular function, ventricular arrhythmic burden, adherence, substance abstinence, and the reversibility of the underlying myocardial injury are reassessed [86]. The device may therefore serve as a bridge either to recovery, allowing permanent device implantation to be avoided, or to implantable cardioverter-defibrillator implantation when persistent high-risk features remain [86]. An updated meta-analysis including 40 studies and 59,647 patients reported an overall appropriate wearable cardioverter-defibrillator intervention rate of approximately 3%, with the greatest arrhythmic vulnerability occurring during the early follow-up period and benefit being strongly dependent on appropriate patient selection and adherence [87]. Nevertheless, wearable cardioverter-defibrillator use should be individualized through multiparametric assessment rather than routinely prescribed to all patients with drug-associated ACS.

#### 6.2.4. Emergency Department Evaluation and Toxicology-Positive Patients

Emergency department evaluation of suspected drug-induced ACS presents several diagnostic and therapeutic challenges. Although patients should generally be managed according to standard ACS protocols, recognition of recent stimulant use may influence acute treatment decisions. A detailed substance-use history should be obtained whenever possible; however, underreporting is common, and toxicology screening may provide valuable information in selected individuals, particularly younger patients, those without traditional cardiovascular risk factors, or those with clinically suspected substance use. This approach is supported by the ADDICT-ICCU study, in which more than half of patients with positive toxicology results had not disclosed recreational drug use [15].

Cocaine- and methamphetamine-associated presentations may mimic conventional ACS but can also reflect coronary vasospasm, severe hypertension, or aortic dissection, requiring a broad differential diagnosis and careful clinical assessment [5,73]. Patients with suspected stimulant-related chest pain should undergo standard ACS evaluation, including serial electrocardiograms and cardiac biomarker measurements, while management should also address sympathetic overstimulation when present. These considerations underscore the importance of maintaining a high index of suspicion for substance use in selected patients presenting with acute chest pain.

## 7. Preventive Strategies

### 7.1. Public Health Approaches

Large, well-designed studies are needed to better define the long-term cardiovascular effects of recreational drug use, particularly on atherosclerosis progression and mortality [88,89]. A better understanding of the complex pathophysiological mechanisms of these substances is essential for clinicians, as it may improve recognition of the broad spectrum of clinical manifestations associated with both acute and chronic use [88]. Public health services should ensure adequate treatment capacity for people with recreational drug use and develop structured assessment models [90]. Examples of potentially adaptable interventions include prescription drug monitoring programmes, community-based harm-reduction services, overdose-prevention education, take-home naloxone distribution, linkage-to-care pathways after emergency presentations, and targeted screening or counseling programmes for high-risk populations. Within cardiovascular prevention, these strategies could be adapted to facilitate earlier identification of substance use, improve referral to addiction services, support treatment adherence, and reduce recurrent cardiovascular events. Early referral to substance use treatment specialists is important for timely initiation of appropriate interventions for stimulant use disorder [91]. Take-home naloxone programmes, primarily implemented to reduce overdose-related mortality, are also relevant to cardiovascular care because reversal of opioid-induced respiratory depression and hypoxemia may prevent secondary myocardial injury, arrhythmias, and cardiac arrest. However, naloxone administration may precipitate acute withdrawal and catecholaminergic activation; therefore, patients with chest pain, arrhythmias, hemodynamic instability, or underlying cardiovascular disease require appropriate monitoring after reversal [92,93].

### 7.2. Screening and Early Intervention in High-Risk Populations

Recreational drug use is common in young ACS patients and has been associated with larger MI and more advanced cardiac dysfunction, highlighting the need for increased awareness and tailored management [94]. Drug use should therefore be included in history-taking and risk stratification of patients presenting with symptoms or signs of ACS [15]. However, a significant proportion of young ACS patients do not have documented recreational drug history or toxicology screening, suggesting under-recognition by physicians [94]. Thorough drug history and selected toxicology screening may improve risk assessment, management, and counseling, particularly in young patients, those with few traditional risk factors, or those presenting with unexplained STEMI, NSTEMI, arrhythmias, or cardiac arrest [15,94]. Binge stimulant use, concurrent alcohol or central nervous system depressant use, overdose history, and stimulant-related emergency department visits or hospitalizations should also be assessed to identify high-risk individuals [90]. Current ACS risk stratification scores do not include recreational drug use, underscoring the need for tailored approaches [94]. In this context, systematic urine or plasma toxicology screening in STEMI patients, especially those younger than 50 years, may be considered to identify patients at higher risk of cardiovascular complications [95].

Toxicology testing should be interpreted cautiously and always in the context of the clinical presentation. A positive urine result generally confirms previous exposure within a substance-specific detection window but does not establish the timing of use, acute intoxication, or a causal relationship with the cardiovascular presentation. Conversely, a negative result does not exclude recent exposure, particularly when presentation is delayed, the detection window is short, the substance or metabolite is not included in the standard panel, or the assay has limited analytical sensitivity. Detection characteristics differ substantially among substances: cocaine metabolites are generally detectable for a limited period after use, cannabinoids may remain detectable for considerably longer—particularly after frequent or chronic exposure—and opioids and amphetamines vary according to the specific compound, dose, route, metabolism, and timing of sampling [96]. Standard immunoassays may also fail to identify many synthetic cannabinoids, novel psychoactive substances, fentanyl analogues, or adulterants, while cross-reactivity with prescribed or over-the-counter medications may produce false-positive results. Immunoassay-positive results should therefore be considered presumptive when the result is unexpected or clinically consequential, with confirmation using a more specific analytical method such as gas or liquid chromatography coupled with mass spectrometry [96,97]. Toxicology findings should support, rather than replace, careful history-taking, electrocardiographic and biomarker assessment, imaging, and clinical judgment. Routine indiscriminate screening may also increase costs and raise concerns regarding stigmatization, confidentiality, insurance, or employment.

### 7.3. Patient Education and Harm Reduction

Given the wide range of adverse cardiovascular outcomes linked to recreational drugs, patient education regarding potential cardiac risks is essential, together with access to appropriate treatment strategies for individuals with ongoing drug use [88]. Continued counseling is also important, as a substantial proportion of patients with recreational drug use presenting with ACS continue drug use after discharge [98]. Avoidance of poly-drug use and modification of additional lifestyle factors, including concurrent alcohol consumption and tobacco use, are particularly relevant because these behaviours may further increase cardiovascular risk and contribute to poorer outcomes [15,88]. Ongoing monitoring and management of heart rate and blood pressure abnormalities may help prevent complications related to untreated stimulant-induced sympathomimetic hyperactivity. In patients with recent stimulant use presenting with chest pain, the use of a coronary vasodilator, such as nitroglycerine or a calcium channel blocker, together with a gamma-aminobutyric acid-ergic (GABA) agent, such as benzodiazepine, may be considered when no contraindications exist.

Psychosocial interventions, including motivational interviewing, cognitive behavioural therapy, and contingency management, may support reduction or cessation of substance use and improve engagement with treatment, particularly in stimulant and cocaine use disorders. Meta-analyses support their role in reducing cocaine use, while family therapy and interpersonal psychotherapy may improve treatment adherence [99]. The Matrix model is also an evidence-based structured approach for stimulant use disorder, incorporating individual therapy, family education, and social support groups to facilitate abstinence [99]. These approaches may also facilitate engagement with cardiovascular follow-up and adherence to evidence-based secondary-prevention therapies after ACS.

## 8. Knowledge Gaps and Future Directions

Despite growing clinical recognition of recreational drug use as a potential trigger of ACS, evidence regarding optimal management and prevention remains scarce. Much of the available evidence comes from case reports, small case series, preclinical studies, postmortem analyses, and retrospective cohorts, while larger observational studies are limited by reliance on self-reported drug use, inconsistent toxicological screening protocols, and frequent polysubstance use.

Important mechanistic uncertainties remain. Although vasospasm, inflammation, accelerated atherosclerosis, sympathomimetic surges, and increased oxygen demand have been proposed as key pathogenetic mechanisms for substances such as cocaine, methamphetamine, and cannabis, most data remain indirect [100,101]. The mechanisms by which methamphetamine promotes local vascular inflammation and vulnerable atherosclerotic plaque formation are incompletely understood [11]. Similarly, the contribution of CB1 activation, oxidative stress, and inflammation to cannabis-induced myocardial ischemia requires further validation in human studies [10].

Long-term cardiovascular outcomes after recreational drug-associated ACS also remain insufficiently defined. A recent multicenter French registry showed an almost threefold increase in major adverse cardiovascular events (MACE) 12 months after the index event among patients with recreational drug use, particularly in those presenting with STEMI [16]. However, the impact of drug use in NSTEMI patients remains underexplored, and the underlying mechanisms may differ from those observed in STEMI. Drug-associated STEMI may more often reflect acute vasospasm, coronary thrombosis, or abrupt plaque disruption, whereas NSTEMI may more frequently occur in the setting of chronic atherosclerotic disease, endothelial dysfunction, microvascular ischemia, or myocardial oxygen supply–demand mismatch. These distinctions remain incompletely defined and require further prospective investigation. Similarly, a retrospective Dutch cohort found that drug-related ACS was associated with larger infarct size, higher cardiac biomarker levels, and lower left ventricular ejection fraction compared with drug-naive ACS in the acute setting, but the mechanisms underlying these findings and their long-term prognostic implications remain unclear [94].

Several therapeutic uncertainties also persist. In cocaine-associated ACS, the role of beta-blockers remains debated. Although earlier concerns focused on possible worsening of alpha-adrenergic stimulation and coronary vasoconstriction, systematic reviews and meta-analyses have not shown a significant increase in MI or all-cause mortality with beta-blocker use, with labetalol showing a favorable safety profile [77,102]. Observational data from the RUTI-Cocaine study also suggested lower mortality and MI among patients treated with beta-blockers during hospitalization and after discharge [78]. However, these data remain non-randomised, and no dedicated randomised controlled trial has clarified the optimal timing, agent, or patient selection for beta-blocker use in this setting. Conducting such trials is challenging because eligible patients often present unpredictably during acute intoxication, may have impaired capacity to provide informed consent, and frequently differ substantially in dose, route, timing, purity of exposure, co-ingested substances, hemodynamic status, and underlying coronary disease. Ethical concerns regarding treatment allocation during active ischemia, together with recruitment difficulties and the relative infrequency of well-characterized cocaine-associated ACS, further complicate randomised study design. The Italian Association of Hospital Cardiologists (ANMCO) position statement has suggested considering carvedilol because of its alpha-blocking activity, although data remain insufficient [4]. In cannabis-associated ACS, β-blocker therapy should be individualized according to haemodynamic status, left ventricular function, conduction abnormalities, arrhythmic indications, and the underlying mechanism of myocardial ischemia or injury, rather than used solely for heart-rate control [103].

Collaborative cardiology–addiction care models may include co-located or closely coordinated cardiology and addiction services, standardized screening and referral pathways, shared treatment protocols, multidisciplinary case review, integrated cardiac rehabilitation, behavioural counseling, medication-assisted treatment when indicated, and shared access to relevant clinical information through the electronic health record [5]. Such models may improve continuity of care, treatment engagement, adherence to secondary prevention, and follow-up after discharge; however, direct evidence demonstrating reductions in recurrent ACS or mortality remains limited. Behavioural cardiology services may also provide structured support for risk-factor modification, treatment adherence, and substance-use reduction within a multidisciplinary framework [104]. Future studies should therefore use standardized toxicology protocols, distinguish between specific substances and polysubstance use, assess long-term outcomes, and test integrated cardiology–addiction care pathways in prospective cohorts and randomised trials.

## 9. Conclusions

Recreational drugs should not be regarded as incidental findings in patients presenting with acute coronary syndrome. Cannabis, cocaine, amphetamines, MDMA, opioids, and other psychoactive agents may act as clinically relevant triggers of myocardial ischemia and infarction through overlapping mechanisms, including coronary vasospasm, thrombosis, sympathetic activation, oxygen supply–demand mismatch, endothelial dysfunction, inflammation, arrhythmogenesis, and accelerated atherosclerosis. Drug-related ACS often affects younger individuals with fewer traditional cardiovascular risk factors and may present with STEMI, arrhythmias, cardiac arrest, or recurrent adverse events. Clinically, drug-related ACS should be actively considered in young patients, in those with unexplained STEMI or NSTEMI, coronary vasospasm, arrhythmias, cardiac arrest, or findings disproportionate to traditional risk factor burden. Because recreational drug use is frequently underreported, targeted toxicology screening may improve diagnostic accuracy, risk stratification, and individualized management in selected high-risk patients.

Future research should focus on prospective studies with standardized toxicology protocols, substance-specific risk assessment, and dedicated treatment pathways. Long-term care should integrate cardiology, addiction medicine, cardiac rehabilitation, and behavioural cardiology to reduce recurrent events, support abstinence, and address recreational drug use as both an ACS trigger and a marker of adverse prognosis.

## Figures and Tables

**Table 1:** Differential diagnosis and management of acute myocardial injury after recreational drug exposure.

Clinical Entity	Main Mechanism	Typical Angiographic Findings	Key Imaging/Diagnostic Clues	Management Implication
Type 1 MI	Plaque rupture/erosion with thrombosis	Obstructive culprit lesion or acute occlusion	Regional wall-motion abnormality; OCT/IVUS may show plaque disruption or thrombus	Standard ACS therapy ± PCI
Coronary thrombosis without major atherosclerosis	Platelet activation, endothelial dysfunction, or hypercoagulability	Thrombus with minimal fixed stenosis	OCT/IVUS may exclude occult plaque disruption or dissection	Individualized antithrombotic therapy ± PCI
Epicardial vasospasm or coronary microvascular dysfunction	Transient vasoconstriction or endothelial dysfunction	Normal or non-obstructive coronary arteries; reversible epicardial narrowing may be seen with vasospasm	Transient ischemic changes; functional testing in selected stable patients	Nitrates/CCBs and trigger avoidance
Type 2 MI	Oxygen supply–demand imbalance	No acute atherothrombotic culprit lesion	Troponin rise/fall with evidence of ischemia; regional or global dysfunction may occur	Correct the underlying imbalance
Myocarditis or direct toxic injury	Inflammation or direct cardiotoxicity	Unobstructed arteries	CMR: oedema and non-ischemic injury pattern	Disease-specific/supportive treatment
Takotsubo syndrome	Catecholamine-mediated myocardial stunning	Unobstructed arteries	Dysfunction beyond one coronary territory; CMR excludes myocarditis/MI	Supportive care and reassessment
SCAD	Intramural hematoma or intimal disruption	Long smooth narrowing or dissection pattern	OCT/IVUS selectively when uncertain	Conservative management when stable

ACS, acute coronary syndrome; CCBs, calcium-channel blockers; CMR, cardiac magnetic resonance; IVUS, intravascular ultrasound; MI, myocardial infarction; OCT, optical coherence tomography; PCI, percutaneous coronary intervention; SCAD, spontaneous coronary artery dissection.

**Table 2:** Guideline-based recommendations relevant to drug-associated ACS.

Source	Clinical Context	Main Recommendation or Practical Implication
ACC/AHA/ACEP/NAEMSP/SCAI/ACS 2025 Guidelines [2]	General ACS management	Standard ACS pharmacotherapy, reperfusion strategy, invasive assessment, and secondary prevention should be applied according to ACS presentation and risk profile.
ESC ACS Guidelines 2023 [1]	General ACS management	Standard ACS treatment, invasive strategy, antithrombotic therapy, and secondary prevention remain the foundation of care.
AHA Scientific Statement 2008 [5]	Cocaine-associated chest pain/MI	Benzodiazepines, nitrates, antiplatelet and antithrombotic therapy, and PCI when indicated; careful assessment for vasospasm and complications.
AHA/ACC NSTE-ACS Guidelines 2014 [73]	NSTE-ACS with suspected stimulant use	Avoid acute β-blocker administration in patients with signs of active cocaine intoxication because of concern for worsening vasospasm.

ACC, American College of Cardiology; ACS, acute coronary syndrome; AHA, American Heart Association; ACEP, American College of Emergency Physicians; MI, myocardial infarction; NAEMSP, National Association of EMS Physicians; NSTE-ACS, non-ST-elevation acute coronary syndrome; PCI, percutaneous coronary intervention; SCAI, Society for Cardiovascular Angiography and Interventions.

**Table 3:** Practical acute management considerations in drug-associated ACS.

Intervention	Standard ACS	Cocaine-Associated ACS	Methamphetamine-Associated ACS
Primary PCI/invasive strategy	Recommended according to ACS type and risk profile	Recommended when indicated; preferred reperfusion strategy in STEMI when available	Recommended when indicated; manage as ACS while addressing stimulant toxicity
Dual antiplatelet therapy	Recommended	Recommended	Recommended
Anticoagulation	Recommended during acute management	Recommended according to standard ACS protocols	Recommended according to standard ACS protocols
Nitrates	Recommended for ongoing ischemic symptoms when appropriate	Particularly useful for coronary vasospasm and hypertension	Useful for ischemia, vasospasm, or hypertension
Benzodiazepines	Not routine in standard ACS	Recommended when anxiety, hypertension, tachycardia, or sympathetic excess is present	Recommended when agitation, hypertension, tachycardia, or sympathetic excess is present
β-blockers	Selective use in haemodynamically stable patients without contraindications	Avoid during acute intoxication; may be considered later in selected patients, especially with α/β-blocking agents when clinically appropriate	Generally, avoid during acute intoxication; evidence is limited and largely extrapolated from cocaine-associated ACS
Calcium channel blockers	Selective use	Consider when ischemic symptoms or vasospasm persist despite nitrates and benzodiazepines	Consider for persistent vasospasm or hypertension when clinically appropriate
Fibrinolysis	Reserved for STEMI when timely PCI is unavailable	Use with caution because severe hypertension, trauma, seizures, or aortic dissection may increase bleeding risk	Use with caution; assess for contraindications and complications of stimulant toxicity

ACS, acute coronary syndrome; DAPT, dual antiplatelet therapy; PCI, percutaneous coronary intervention; STEMI, ST-segment elevation myocardial infarction.

## Data Availability

No new data were created or analyzed in this study. Data sharing is not applicable to this article.

## Abbreviations

ACC	American College of Cardiology
ACEP	American College of Emergency Physicians
ACS	Acute coronary syndrome
AHA	American Heart Association
CAD	Coronary artery disease
CB1	Cannabinoid receptor type 1
CB2	Cannabinoid receptor type 2
CI	Confidence interval
CNS	Central nervous system
CVD	Cardiovascular disease
DAPT	Dual antiplatelet therapy
DOR	Delta-opioid receptor
ECG	Electrocardiogram
EMS	Emergency medical services
ESC	European Society of Cardiology
HR	Hazard ratio
ICCU	Intensive cardiac care unit
KOR	Kappa-opioid receptor
LDL-C	Low-density lipoprotein cholesterol
LSD	Lysergic acid diethylamide
MACE	Major adverse cardiovascular events
MDMA	3,4-methylenedioxymethamphetamine
MI	Myocardial infarction
MOR	Mu-opioid receptor
NAEMSP	National Association of EMS Physicians
NSTEMI	Non-ST-segment elevation myocardial infarction
NSTE-ACS	Non-ST-elevation acute coronary syndrome
OR	Odds ratio
PCI	Percutaneous coronary intervention
PCSK9	Proprotein convertase subtilisin/kexin type 9
P2Y12	P2Y12 receptor
ROS	Reactive oxygen species
SCAI	Society for Cardiovascular Angiography and Interventions
STEMI	ST-segment elevation myocardial infarction
THC	Δ9-tetrahydrocannabinol
